# COVID-19 pandemic and mental healthcare: Impact on health insurance with guaranteed universal access in Chile

**DOI:** 10.3389/fpubh.2022.1005033

**Published:** 2023-01-04

**Authors:** Olga Toro-Devia, Gonzalo Leyton

**Affiliations:** ^1^School of Public Health, Faculty of Medicine, Universidad de Chile, Santiago, Chile; ^2^Doctoral Program in Public Health, University of Chile, Santiago, Chile; ^3^Superintendence of Health, Studies Department, Santiago, Chile

**Keywords:** mental health, universal coverage, COVID-19 pandemic, health insurance, financing

## Abstract

**Background:**

Universal health coverage (UHC) is a goal of the member states of the United Nations. The negative impact of the COVID-19 pandemic on mental health, inequalities in access to care, and financing gaps set a problematic scenario for universal mental health coverage. In Latin America, depression and anxiety disorders have increased by more than 30%. Chile implemented a reform for UHC in 2005 generating a mandatory guaranteed plan for health insurance (GES) that covers schizophrenia, depression, bipolar disorders, and Alzheimer's disease. We assume that the pandemic increased cases of mental illness in GES of public and private insurance.

**Objectives:**

This study aimed to explore the effects of the pandemic on the use of the GES mental health plan of public and private insurance.

**Methods:**

A descriptive analysis of secondary data from public and private insurance on the use and expenditure of the GES plan in mental illness between 2005 and 2020 was carried out. An aggregate analysis of the use of psychiatric consultations without a guaranteed plan and sick leave was performed.

**Results:**

Between 2005 and 2020, 18.5% of GES cases corresponded to four mental health illnesses (1,682,021 cases). Public insurance covered 80% of cases. In the pandemic, cases of mental illness fell by 10.5% in public insurance and 28.7% in private ones, reducing spending by 33 and 6.2%, respectively. Psychiatric consultations without using the GES plan doubled in 2020 in private insurance, and medical discharges due to mental illness also increased. Leave due to mental illness increased by 20% in both types of insurance.

**Conclusion:**

The results suggest that the demand for mental healthcare increased during the pandemic, but public and private health insurance reduced admissions to the GES universal plan for schizophrenia, depression, and bipolar disorder. A universal guaranteed plan in an individual contribution system can have essential weaknesses for people when the principles of social security are not complied with, especially concerning the solidarity of the health insurance system.

## 1. Introduction

Before the COVID-19 pandemic in the Americas, mental, substance use, neurological disorders, and suicide accounted for one-third of all years lived with disability (YLD) and one-fifth of total disability-adjusted life years (DALYs) (1). Depression and anxiety disorders were among the 25 diseases with the highest DALYs, while deaths from drug use disorders have risen over the past decade (2, 3). The first cases of COVID-19 were identified in December 2019 in the Chinese city of Wuhan. The World Health Organization declared it a public health emergency of international concern on 30 January 2020 and recognized it as a pandemic on 11 March 2020. The first case of the COVID-19 pandemic in Chile was confirmed on 3 March 2020. The outbreak of the pandemic is exacerbating the mental health landscape. The Americas is the most affected by the pandemic, with 39% of infected cases and 46% of all deaths worldwide (September 2021) (4). It is estimated that in Latin America and the Caribbean, the prevalence of depression and anxiety disorders increased by more than 30% (5). The region of the Americas has profound inequalities in response to mental health services, and there is an imbalance between the burden of mental illness and the health budget allocated to mental health (6).

The Member States of the United Nations have committed to attempting to achieve universal health coverage by 2030 at the latest, within the framework of the achievement of the Sustainable Development Goals (3, 7). The 74th World Health Assembly (May 2021) recognized the importance of expanding access to mental health services; the World Health Organization called on all countries to make substantial investments in mental health as part of their journey toward universal health coverage. The negative impact of the pandemic on mental health, inequalities in access to mental healthcare, and financing gaps make up a problematic scenario for universal mental health coverage in the region.

Chile implemented a reform for universal health coverage in 2005, based on the profound inequities in the healthcare of its population. This reform generated a mandatory guaranteed plan for public (FONASA) and private (ISAPRES) health insurance called Universal Plan with Explicit Guarantees in Health (GES) (8). The GES plan establishes legal guarantees of access, opportunity, quality, and financial protection. It regulates the maximum price that a person can co-pay for each health problem in both types of insurance. A total of 78% of the population is affiliated with public insurance, while 18% with private insurance. The GES plan began with 25 health problems, which increased over the years. Currently, the GES plan covers 85 health problems, among those that are linked to mental disorders are as follows: schizophrenia, depression, bipolar disorders, and Alzheimer's disease, called “GES mental health plan” in this study. It has been argued that the principle of universal coverage of the GES plan has contributed to access to care for schizophrenia, especially for the most vulnerable population (9).

However, the pandemic had adverse effects on mental health services in Chile since they significantly reduced their capacity for care, especially in the first months. The country made a significant effort to develop strategies to address mental health needs during the pandemic (10). Examples of this effort are the “Saludablemente” plan to strengthen mental healthcare measures during a pandemic and the new law 21,331 to protect mental healthcare, which requires parity between mental health and other health problems (11). Despite public efforts, the effect on access to mental healthcare has not been evaluated, considering a universal guaranteed plan.

The objective of our study is to explore the effects of the pandemic on the use of the GES mental health plan of public and private insurance. In this context, the study is relevant as the effects of COVID-19 on mental health are compounded by the current global economic crisis, where the latest available evidence (12) shows that a change in income is followed by a subsequent change in wellbeing and mental health, suggesting a unidirectional causal effect of income on mental health and wellbeing. Our purpose is to provide evidence for decision-makers who will face the dilemma of how to continue advancing the commitments of universal coverage in mental health in an adverse scenario. Although the study is limited to only one Latin American country, Chile is interesting for other countries because it has a universal access plan for public and private health insurance, including mental health problems. The GES plan would mean progress toward universal health coverage.

## 2. Materials and methods

### 2.1. Data sources and study design

It is an observational study based on secondary data. Affiliates to public health insurance are around 15 million people and private health insurance is 3 million people.

### 2.2. Study variables and samples

We collected data on the use of the GES plan for schizophrenia, depression, bipolar disorder, and Alzheimer's disease reported by public insurance and private insurance between 2005 and 2020, which accumulates a total of 1,682,021 cases. Legal regulations oblige them to report to a government health entity called the Superintendence of Health. The data are anonymized. We included data on new case intakes and monetary insurance expenses using the plan. Based on the preliminary results, we consider it necessary to corroborate whether the demand for mental healthcare has increased since the pandemic, without the use of the GES plan. Because people can accept or reject care for their health problems with the GES plan, we collected data on a) consultations and teleconsultations with a psychiatrist for mental health problems without using the GES plan (it was possible for private insurance); and b) sick leave granted for any mental health diagnosis according to the ICD-11 classification (Mental, Behavioral and Neurodevelopmental Disorders Group) of 2019 and 2020.

### 2.3. Data analysis

We analyzed data descriptively. We calculated the frequencies and percentages of new cases according to the type of insurance, mental health problem, and year. We estimated the proportion of use due to mental health problems about the full use of the GES plan. The use ratio was standardized according to the population effectively affiliated with the insurance, and the public/private use rate ratio was estimated. We calculated the variation in use ratio over the years for schizophrenia, depression, and bipolar disorder. We detailed the comparison between 2019 and 2020. Data on Alzheimer's disease are limited because the law incorporated it into the GES plan in 2019.

The proportion of spending in the GES plan that went to mental health problems by the type of insurance was calculated. We estimated the variation in this expense before and after the start of the pandemic.

We reported that consultations and teleconsultations with a psychiatrist for mental health problems without using the GES plan in private insurance have increased. We checked whether the mental health sick leaves had increased. Only the doctor prescribes sick leave, and there was a greater demand for medical consultation, even if people did not use their guaranteed plan (GES). Chile guarantees paid sick leave to all its workers. We compared the total number of sick leave processed with the proportion of those that got rejected or reduced the prescribed rest days by insurance.

## 3. Results

Since the GES plan began in 2005, 18.5% of the cases correspond to mental health. Of the total GES cases of private insurance, 15.1% of the cases correspond to mental health. Of the total GES cases of public insurance, 3.4% of the cases correspond to mental health (Table 1). Although public insurance shows relatively fewer mental health cases than private insurance, it covered 80.1% of the total mental health cases when both insurances were added. This is because public insurance covers most of the population, with more than 40 million GES cases in the entire period vs. a little more than 2 million GES cases from private insurance.

**Table 1:** Mental disorders in universal health guarantee plan GES.

**Measure**	**Cumulative cases** **Jul 2005–Dec 2020**		**New cases** **Jan–Dec 2019**		**New cases** **Jan–Dec 2020**	
	**Public insurance**	**Private insurance**	**Public insurance**	**Private insurance**	**Public insurance**	**Private insurance**
Total GES cases (*N*) mental disorders	1,347,726	334,295	60,790	24,498	54,405	17,466
Total GES cases (change %) mental disorders					−10.5%	−28.7%
Total GES cases (*N*)	40,112,042	2,212,441	3,396,714	194,535	1,980,456	132,694
Total GES cases (change %)					−41.7%	−31.8%
Total GES cases (*N*) mental disorders/Total GES cases (*N*)	3.4%	15.1%	1.8%	12.6%	2.7%	13.2%
Total GES expenditure (US$) mental disorders			36,636,174	35,084,002	24,441,205	32,922,125
Total GES expenditure (real change %) mental disorders					−33.3%	−6.2%
Total GES expenditure (US$)^*^, ^**^			761,827,087	310,734,773	643,792,382	296,556,252
Total GES expenditure (change %)					−15.5%	−4.6%
Total GES expenditure (US$) mental disorders/Total GES expenditure (US$)			4.8%	11.3%	3.8%	11.1%

* Excluding co–payments;

** Exchange rate: 1 US$ = 792.2 pesos chilenos.

In the first year of the pandemic, mental health cases in public insurance decreased by 10.5% compared to 2019. This number is relatively small compared to the total decrease in cases of the GES plan of 41.7% for that insurance. In contrast, in private insurance, mental health cases decreased between 2019 and 2020 by 28.7%, a figure close to the total reduction of their GES cases of 31.8%.

Although in 2019 the number of cases in public insurance were more than twice as high as those in private insurance, the expense spending on mental health differed by just 4.4% between both insurances. In 2019, mental health spending represented 4.8% of the total GES plan in public insurance and 11.3% in private insurance. In 2020, the mental health expense would be 33.3% in public insurance, proportionally double the total expense of your GES plan will owe (−15.5%). In private insurance, the decrease in spending on mental health was 6.2%, a relative figure higher than the 4.6% decrease in spending for the total of its GES plan. In the first year of the pandemic, the expenditure of the GES plan on the mental health of public insurance was 26% below that of private insurance, although its cases were three times higher.

Compared between 2019 and 2020, schizophrenia had an annual rate of 22.5 cases vs. 17.1 per 100,000 members of public insurance and 9.1 vs. 8.2 in private insurance. The public/private ratio of cases x 100,000 affiliates is 2.5 in 2019 and dropped to 2.1 in 2020. Therefore, the rate of public use is more than double that of private insurance. Despite this difference, private spending for schizophrenia represented 0.9 times public spending in 2019, while private spending was 1.2 times higher than public spending in the first year of the pandemic. Public insurance decreased its GES expenditure for schizophrenia by 23.9% compared to both years. It should be noted that in public insurance, cases of schizophrenia represent 3.1% of the accumulated cases in the entire period, 5.1% in 2019, and 4.4% in 2020. While for private insurance, the accumulated is 1.2%, for 2019 is 1.3%, and for 2020 is 1.6%.

Of the four mental health problems included in the GES plan, cases of depression in 2019 represented more than 85% (Table 2). This proportion changed in the first year of the pandemic, particularly in public insurance, which fell to 62.3%, although they continue to be the most prevalent cases. Between 2019 and 2020, depression had an annual rate of 464.3 cases vs. 304.1 per 100,000 members of public insurance and 783.6 vs. 530.5 in private insurance. The public/private ratio of cases x 100,000 affiliates is 0.6 for both years. Therefore, the rate of private use is much higher than public use. Private spending for depression was 0.7 times public spending in 2019, while in 2020, they spent practically the same. However, of the total cases of GES depression, only 30% corresponded to private insurance. Public insurance decreased its GES expense for depression by 34.5% compared to both years.

**Table 2:** Mental disorders in the universal health guarantee plan GES according to the type of diagnosis.

**Mental disorders**	**Measure**	**Cumulative cases** **Jul 2005–Dec 2020**		**New cases** **Jan–Dec 2019**		**New cases** **Jan–Dec 2020**	
		**Public insurance**	**Private insurance**	**Public insurance**	**Private insurance**	**Public insurance**	**Private insurance**
Schizophrenia	Cases (*N*)	41,151	4,111	3,112	310	2,369	282
	Cases (%)	(3.1%)	(1.2%)	(5.1%)	(1.3%)	(4.4%)	(1.6%)
	Total annual use rate^*^			22.5	9.1	17.1	8.2
	Total annual use rate (change %)					−24.0%	−9.9%
	Public/Private use rate ratio			2.5		2.1	
	GES expenditure (US$)^**^, ^***^			3,848,431	3,388,499	2,929,605	3,548,167
	GES expenditure (%)			(10.5%)	(9.7%)	(12.0%)	(10.8%)
	GES expenditure (2020–2019)–change %					−23.9%	4.7%
Depression (aged 15 +)	Cases (*N*)	1,262,916	304,852	51,758	21,048	33,896	14,249
	Cases (%)	(93.7%)	(91.2%)	(85.1%)	(85.9%)	(62.3%)	(81.6%)
	Total annual use rate^*^			464.3	783.6	304.1	530.5
	Total annual use rate (change %)					−34.5%	−32.3%
	Public/Private use rate ratio			0.6		0.6	
	GES expenditure (US$)^**^, ^***^			31,056,659	22,763,871	20,338,818	20,937,896
	GES expenditure (%)			(84.8%)	(64.9%)	(83.2%)	(63.6%)
	GES expenditure (2020–2019)–change %					−34.5%	−8.0%
Bipolar disorders	Cases (*N*)	23,786	24,181	2,496	2,714	1,691	2,210
(aged 15 +)	Cases (%)	(1.8%)	(7.2%)	(4.1%)	(11.1%)	(3.1%)	(12.7%)
	Total annual use rate^*^			22.4	101	15.2	82.3
	Total annual use rate (change %)					−32.1%	−18.5%
	Public/Private use rate ratio			0.2		0.2	
	GES expenditure (US$)^**^, ^***^			1,731,084	8,931,632	1,172,782	8,436,062
	GES expenditure (%)			(4.7%)	(25.5%)	(4.8%)	(25.6%)
	GES expenditure (2020–2019)–change %					−32.3%	−5.5%
Alzheimer's disease and	Cases (*N*)	19,873	1,151	3,424	426	16,449	725
other dementias	Cases (%)	(1.5%)	(0.3%)	(5.6%)	(1.7%)	(30.2%)	(4.2%)
	Total annual use rate^*^			41.4	21.5	199	36.5
	Total annual use rate (change %)					380.7%	69.8%
	Public/Private use rate ratio			1.9		5.4	
	GES expenditure (US$)^**^, ^***^			1,202,908	20,355	5,778,806	251,113
	GES expenditure (%)			(3.3%)	(0.1%)	(23.6%)	(0.8%)
	GES expenditure (2020–2019)–change %					380.4%	1133.7%

* Ratio between GES cases (N) of the target population defined in the Access Guarantee of the Supreme Decree in force per 100,000 beneficiaries.

** Excluding co–payments.

*** Exchange rate: 1 US$ = 792.2 pesos chilenos.

For bipolar disorder, compared to 2019 and 2020, public insurance had a rate of 22.4 and 15.2 cases, respectively, per 100,000 affiliates. As for private insurance, it was 101 and 82.3, respectively. With regard to the cases accumulated throughout the period, cases of bipolar disorder represent 1.8% of public insurance and 7.2% of private insurance. This health problem was incorporated into the GES plan only in 2013. In 2019, they represented 4.1% of public insurance and 11.1% of private insurance. As for 2020, those figures are 3.1 and 12.7%, respectively. The public/private use ratio in both years is 0.2. Private insurers spent more than a quarter of their spending on cases of bipolar disorder.

In comparison, public insurance does not exceed 5% of spending. Overall, even though raw bipolar disorder numbers are slightly lower for public insurance, private insurance spent 5.2 times more in 2019 and 7.2 times more in 2020. Public insurance decreased its GES spending for bipolar disorder by 32.3% compared to both years.

Alzheimer's disease and other dementias were incorporated at the end of 2019 into the GES plan. Therefore, it is expected to observe exponential growth for the first effective year of the plan in 2020. During the first year of the pandemic, the ratio of public/private use was 5.4. Public insurance attended 96% of these GES cases.

Contrary to the study's assumption, the use of the GES plan decreased in the first year of the pandemic. This occurred for the GES plan globally for both public and private insurance. In the case of mental health, this decrease is noteworthy as evidence indicates that the context of the pandemic has had a negative impact on the mental health of the population, increasing the prevalence of mental illnesses, particularly depression. Therefore, it was feasible to assume that the demand for mental healthcare would increase using the plan with universally guaranteed rights. Given these results, we decided to explore the data on psychiatric and psychological care included in the complementary health plan; that is, the GES does not cover it. We were only able to access data from private insurance.

In addition, we review the data on medical leaves for mental illness, comparing 2019 and 2020 for contributors to both insurances. These two types of data allow us to corroborate whether there was an impact on the decrease in the demand for mental healthcare during the pandemic.

Adult psychiatric consultations in private insurance without using the guaranteed plan (GES) were more than twice as high as in 2020, as did the expenditure corresponding to this benefit (Table 3). Psychiatric consultations for children increased by 61 and 53% of spending. In addition, new telemedicine benefits were incorporated (adult and child psychiatric consultation, psychological consultation).

**Table 3:** Psychiatric consultations reported by private insurance without using the GES universal access plan.

**Fonasa MLE code**	**Detail**	**2020**		**2019**		**Insurer expenditure (change %)**	**Frequency–change %**
		Insurer expenditure (US$)^*^	**Frequency (*N*)**	Insurer expenditure (US$)^*^	**Frequency (*N*)**		
0101212	Medical consultation specializing in adult psychiatry	4,031,504	169,713	1,926,297	82,732	109%	105%
0101213	Medical consultation specializing in pediatric and adolescent psychiatry	179,722	7,337	117,800	4,544	53%	61%
0108212	Telemedical consultation specializing in adult psychiatry (1st consultancy)	467,194	15,869				
0108213	Telemedical consultation specializing in pediatric and adolescent psychiatry (1st consultancy)	5,233	248				
0908101	Telerehabilitation: clinical psychologist (45' sessions)	248,743	10,138				

* Exchange rate: 1 US$ = 792.2 pesos chilenos. The grey shade color is the porcentual change between 2019 and 2020.

Sick leave issued for mental illnesses increased by around 20% in both types of insurance (Table 4). In 2019, these represented 24.7% of medical licenses in public insurance and 20.3% in private insurance. In 2020, this proportion increased to 28.6% in public insurance and 28.8% in private insurance. The ratio of public/private medical leave due to mental illness was 1.32 in 2019 and 1.29 in 2020. In other words, both the care data without GES and medical leave point to the fact that there was a significant increase in the demand for care in the case of mental health during a pandemic.

**Table 4:** Leave due to mental illness 2019–2020.

**Sick leave status**	**Diagnosis**	**Measures**	**2019**		**2020**	
			**Public insurance**	**Private insurance**	**Public insurance**	**Private insurance**
Processed	Mental disorders	*N*	1,127,470	322,313	1,344,444	385,819
		%	24.7%	20.3%	28.6%	28.8%
		Sick leaves rate^*^	24	18	29	23
		Public/Private sick leaves rate ratio	1.32		1.29	
		Sick leaves rate (change %)			19.9%	22.9%
	Total processed sick leaves^**^		**4,560,459**	**1,588,311**	**4,695,591**	**1,338,494**
Authorized^***^	Mental disorders	*N*	1,006,447	294,794	1,121,980	313,392
		%	23.3%	14.6%	26.0%	19.6%
		Sick leaves rate^*^	22	17	24	18
		Public/Private sick leaves rate ratio	1.29		1.33	
		Sick leaves rate (change %)			12.1%	9.1%
	**Total authorized sick leaves**		**4.311,033**	**2.016,165**	**4.310,301**	**1.602,393**
Refused	Mental disorders	*N*	119,896	121,565	221,241	176,752
		%	48.8%	56.4%	58.4%	65.6%
		Sick leaves rate^*^	3	7	5	10
		Public/Private sick leaves rate ratio	0.37		0.46	
		Sick leaves rate (change %)			85.6%	49.3%
	**Total refused sick leaves**		**245,925**	**215,351**	**378,719**	**269,521**
Reduced	Mental disorders	*N*	N/I	65,697	N/I	67,848
		%		43.9%		46.8%
		Reduced sick leaves (N)/Processed sick leaves (*N*) by mental disorders		20.4%		17.6%
		Reduced days (%)		51.5%		53.5%
		Sick leaves rate^*^		3.75		3.98
		Sick leaves rate (change %)				6.0%
	**Total reduced sick leaves**	***N***		**149,786**		**144,821**
		**Reduced sick leaves (** ***N*** **)/ Processed sick leaves (** ***N*** **)**		**9.4%**		**10.8%**

* Rate per 100 contributors according to sick leave status.

** Excluding N/I.

*** For private insurance, the N includes sick leaves claimed and partially or totally accepted in the appeal instances. Bold values indicates the total sick leaves by each sick leaves status.

In the sick leave administration system of Chile, these leaves are reviewed by control bodies that implement each insurance. As a result of this supervision, medical leaves can be authorized, rejected, or reduced in the number of rest days. We observe that in public insurance, 89% of medical licenses were authorized in 2019 and 83% in 2020. In private insurance, it was 91% in 2019 and decreased to 81% in 2020. With regard to all medical licenses rejected, the high proportion corresponding to mental illnesses is striking. In 2019, 48.8% of medical licenses rejected by public insurance were due to mental illness; in 2020, it increased to 58.4%.

Meanwhile, in private insurance, it increased from 56.4 in 2019 to 65.6% in 2020; that is, two out of three licenses rejected were due to mental illness. In the case of private insurance, over 90% of the causes of medical leave rejected in mental health correspond to depression, anxiety, and bipolar disorder, and the highest proportion is rejected for women (Table 5). If we compare 2019 with 2020, public insurance increased its rejection rate by 85.6%, two times as much as private insurance, which increased its rejection by 49.3%.

**Table 5:** Sick leave refused or reduced depending on the type of mental illness diagnosis*.

**Sick leave status**	**Mental disorder**	**Measure**	**2019**	**2020**
Refused	Depression (including dysthymia)	*N*	62,401	79,943
		%	51.3%	43.5%
		% Women	65%	60%
	Anxiety disorders	*N*	49,601	85,046
		%	40.8%	46.3%
		% Women	56%	50%
	Bipolar disorders (including cyclothymia) and mania	*N*	3,526	4,732
		%	2.9%	2.6%
		% Women	65%	62%
	**3 mental disorders (** ***N*** **)/Total mental disorders (** ***N*** **)**		**95.0%**	**92.4%**
Reduced	Anxiety disorders	*N*	37,641	42,943
		%	57.3%	63.3%
		Reduced days (%)	51.2%	53.8%
		% Women	56.1%	48.8%
	Depression (including dysthymia)	*N*	23,838	21,048
		%	36.3%	31.0%
		Reduced days (%)	51.9%	53.1%
		% Women	65.2%	60.4%
	Bipolar disorders (including cyclothymia) and mania	*N*	893	866
		%	1.4%	1.3%
		Reduced days (%)	48.4%	50.8%
		% Women	64.7%	65.6%
	**3 mental disorders (** ***N*** **)/Total mental disorders (** ***N*** **)**		**94.9%**	**95.6%**

* Considers only private insurance. Bold values indicates the weight of the three main mental disorders between all mental disorders.

In the case of private insurance, medical licenses may be partially accepted, which implies that they reduce the number of rest days indicated by the doctor. Close to 95% of the reduced licenses are due to depression, anxiety, and bipolar disorder, and a more significant proportion are women (Table 4). We find that 43.9 and 46.8% of all reduced medical leaves correspond to mental illness in 2019 and 2020, respectively. When reviewing the number of reduced days, we observe that more than half of the rest days indicated are reduced. These data indicate that despite the significant demand for mental healthcare and indications of rest by doctors, in the global system, mental health was less protected than the rest of the diseases, particularly in the first year of the pandemic.

**Figure 1:**
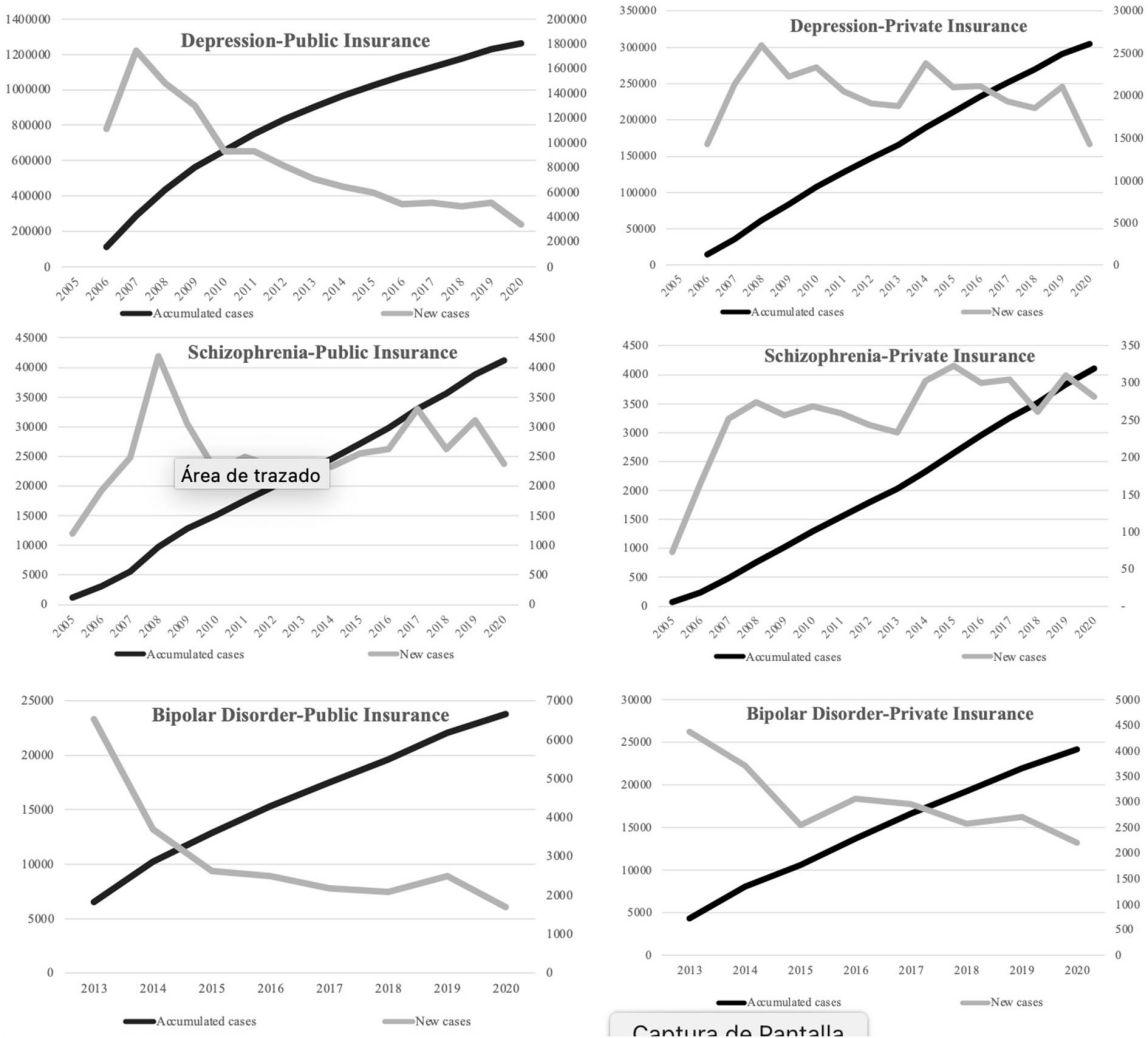
New and accumulated cases per year. Use of the universal health guarantee plan GES in three diagnoses of mental illness according to the type of insurance.

Given these results, we explored the behavior of the rate of use of GES cases since 2005 for the available data in schizophrenia, depression, and bipolar disorder (Figure 1). We observe that in the case of schizophrenia and depression, the first 2 years of incorporating the GES plan tend to increase in cases. Then, there is a variable behavior. For schizophrenia in public insurance from 2010 onwards, relative stability has been maintained at around 2,500 new cases per year. Meanwhile, private insurance has an upward trend in new cases.

In the case of depression, in public insurance from 2007 onwards, a constant decrease in new cases is observed. While in private insurance, there is also a downward trend but not as marked. In both insurances, a more significant decrease is observed in the first year of the pandemic. In the case of bipolar disorder, the first year of incorporation into the GES shows the highest number of new cases. Then, the trend is decreasing for both insurances, although more marked in public.

## 4. Discussion

Contrary to the initial assumption of this study, the results reflect that during the pandemic, public and private health insurance reduced admissions to the GES universal plan for schizophrenia, depression, and bipolar disorder, even though an increase in the prevalence of mental illnesses has been reported (5). One hypothesis is that this decrease can be linked to the reorganization of health care providers that prioritized care for COVID-19, which, contrary to the recommendations (4), implied the closing of access to mental health services, so that the insurance would not have had space to cover the services.

However, the increase in mental health problems was also observed indirectly in the study when it was found that the demand for consultations with a psychiatrist and psychologist without using the GES plan increased, and at the same time, the prescription of mental health sick leave increased. This could indicate that people sought mental healthcare from private providers that were available for care in person or incorporated telemedicine, which they did not provide through the GES plan. It is necessary to investigate why public and private insurance affiliates entered the GES plan less, although it guarantees greater comprehensiveness and quality of care, including financial protection and opportunity.

In contrast, the increase in cases of psychiatric care without GES and sick leave due to mental illness are usually reduced only to medical care without psychosocial intervention, with high co-payments and high rates of rejection of sick leave or a decrease in prescribed rest days. Even so, it seems that in the case of depression and bipolar disorder, the decrease in the use of the GES plan occurred before the pandemic. The opposite is true for schizophrenia, which continued to be used, which may be associated with the fact that, in this case, the GES plan succeeded in improving coverage and financial protection, especially for the most vulnerable (9). The case of increased care for Alzheimer's disease may be associated with the fact that it was the first year of implementation of the GES plan and many people who were already in treatment saw an opportunity to guarantee access and quality of treatment, thanks to the GES plan. This upward trend in the use of the GES plan during the first year of its implementation was also observed in the case of depression and bipolar disorder, although this trend was later reversed.

In contrast, the higher expense observed in the GES plan of private insurance may be associated with the higher prices charged by private providers for the care of the GES plan. The financial protection guarantee of the GES plan ensures a 20% copayment concerning a standard estimated price defined by the supreme decree. However, private providers are free to set prices, charging a price three times higher on average compared to public providers. Private insurance assumes the price difference to guarantee care, and therefore, the financial coverage of the final GES may end up being > 80% (13). However, it must be considered that private insurance continues to receive the GES plan premium from their affiliates even when they do not occupy the GES plan in their care. In public insurance, coverage is 80%, but public providers claim that the decreed prices do not cover the actual cost of care, generating structural debt in the system. When facing these situations, a plan that claims to be universal for all types of insurance ends up not being such.

Countries like Chile, that incorporate the individual health insurance system to advance the universal coverage of mental health (14), have an essential challenge in regulation and control to prevent breaches of guarantees, as well as in resolving how the protection of mental health will be addressed in health emergencies.

## 5. Conclusion

This exploration shows us that people with mental illness had less access to a universal health guarantee plan, alternatively attending individual psychiatric care contrary to a comprehensive model and with less financial coverage, therefore, higher out-of-pocket costs. This may be a problem that is occurring with all types of health insurance (not just the GES plan), and it is necessary to explore whether health insurance systems have been able to cover the mental healthcare needs of their members during the pandemic. In addition, the social protection system for rest indicated by a doctor is more violated in the case of mental illness than in other illnesses. This situation was aggravated in the first year of the COVID-19 pandemic.

The results make it necessary to evaluate the operation of the entire GES plan in the context of the pandemic (not only mental health) since we can learn lessons before continuing to advance the universal mental health coverage policy through this type of plan. A universal guaranteed plan in an individual contribution system can have a significant weakness for individuals when the principles of social security are not complied with, especially regarding the solidarity of the health insurance system. An insurance-based system will require addressing the discussion of reference pricing of mental health actions by providers. Otherwise, universal coverage becomes financially unsustainable.

## Data availability statement

The data analyzed in this study is subject to the following licenses/restrictions. The data comes from the mandatory records of health insurance in the Superintendence of Health (government regulatory entity). The authors must have used the anonymized individual data and we are not authorized to share it. In case of data queries, they should be requested from the corresponding author, who is a researcher at the Superintendence of Health, and it will be possible to share some additional aggregated data if required. Requests to access these datasets should be directed to GL, gonzalo.leyton@gmail.com.

## Author contributions

OT-D and GL discussed the study's introduction, objectives, methods, analyzed the data in the article, organized the tables to summarize the information, jointly analyzed, wrote up the results, discussed the article's conclusions, iterated to summarize the discussion, conclusions, and developed the manuscript. OT-D edited the final version of those chapters. GL edited the final format of the tables. Both authors contributed to the article and approved the submitted version.
